# Physiological response of Symbiodiniaceae to thermal stress: Reactive oxygen species, photosynthesis, and relative cell size

**DOI:** 10.1371/journal.pone.0284717

**Published:** 2023-08-03

**Authors:** Michelle Amario, Lívia Bonetti Villela, Douglas Jardim-Messeder, Arthur Weiss Silva-Lima, Phillipe Magalhães Rosado, Rodrigo Leão de Moura, Gilberto Sachetto-Martins, Ricardo Moreira Chaloub, Paulo Sergio Salomon

**Affiliations:** 1 Laboratório de Fitoplâncton Marinho, Instituto de Biologia, Universidade Federal do Rio de Janeiro, Rio de Janeiro, Rio de Janeiro, Brazil; 2 Programa de Pós-Graduação em Genética, Rio de Janeiro, Instituto de Biologia, Universidade Federal do Rio de Janeiro, Rio de Janeiro, Rio de Janeiro, Brazil; 3 Laboratório de Genômica Funcional e Transdução de Sinal, Instituto de Biologia, Universidade Federal do Rio de Janeiro, Instituto de Biologia, Rio de Janeiro, Rio de Janeiro, Brazil; 4 Laboratório de Biologia Molecular de Plantas, Instituto de Bioquímica Médica, Universidade Federal do Rio de Janeiro, Rio de Janeiro, Rio de Janeiro, Brazil; 5 Red Sea Research Centre, King Abdullah University of Science and Technology, Thuwal, Saudi Arabia; 6 Laboratório de Monitoramento da Biodiversidade, Instituto de Biologia SAGE-COPPE, Universidade Federal do Rio de Janeiro, Rio de Janeiro, Rio de Janeiro, Brazil; 7 Laboratório de Estudos Aplicados em Fotossíntese, Instituto de Química, Universidade Federal do Rio de Janeiro, Rio de Janeiro, Rio de Janeiro, Brazil; University of Innsbruck, AUSTRIA

## Abstract

This study investigates the physiological response to heat stress of three genetically different Symbiodiniaceae strains isolated from the scleractinian coral *Mussismilia braziliensis*, endemic of the Abrolhos Bank, Brazil. Cultures of two *Symbiodinium* sp. and one *Cladocopium* sp. were exposed to a stepwise increase in temperature (2°C every second day) ranging from 26°C (modal temperature in Abrolhos) to 32°C (just above the maximum temperature registered in Abrolhos during the third global bleaching event—TGBE). After the cultures reached their final testing temperature, reactive oxygen species (ROS) production, single cell attributes (relative cell size and chlorophyll fluorescence), and photosynthetic efficiency (effective (Y(II)) and maximum (F_v_/F_m_) quantum yields) were measured within 4 h and 72 h. Non-photochemical coefficient (NPQ) was estimated based on fluorescence values. Population average ROS production was variable across strains and exposure times, reaching up a 2-fold increase at 32°C in one of the *Symbiodinium* sp. strains. A marked intrapopulation difference was observed in ROS production, with 5 to 25% of the cells producing up to 10 times more than the population average, highlighting the importance of single cell approaches to assess population physiology. Average cell size increases at higher temperatures, likely resulting from cell cycle arrest, whereas chlorophyll fluorescence decreased, especially in 4 h, indicating a photoacclimation response. The conditions tested do not seem to have elicited loss of photosynthetic efficiency nor the activation of non-photochemical mechanisms in the cells. Our results unveiled a generalized thermotolerance in three Symbiodiniaceae strains originated from Abrolhos’ corals. Inter and intra-specific variability could be detected, likely reflecting the genetic differences among the strains.

## Introduction

Coral reef ecosystems show a high level of biological interactions complexity, and the success of this environment is commonly attributed to the mutualistic relation between the dinoflagellate of the family Symbiodiniaceae and scleractinian corals [1–3]. The major consequence of this symbiotic relation is the partial autotrophy that the algae allow the coral to benefit from. In this case, Symbiodiniaceae cells recycle the metabolic waste of the cnidarian hosts which are re-metabolized into reusable compounds [4] enhancing coral growth and calcification [5, 6]. In return, the coral provides a protected environment to the symbiont and the nitrogen needed for photosynthesis and growth [7].

The stability of this symbiosis is affected by several factors, some of which have been demonstrated to cause coral bleaching. The disruption of the symbiosis leads to the reduction of the endosymbiont cell density in the cnidarian tissues, pigment content or both. Bleaching can be triggered by temperature (lower or higher than average) [8, 9], high irradiance [10, 11], changes in nutrients and heavy metal concentrations [12] and can be enhanced by the presence of pathogenic micro-organisms [13]. The main factors responsible for large scale coral bleaching events in tropical reefs are elevated sea surface temperature (SST), often combined with high irradiances [14–16]. In high-latitude, extreme environments, and some marginal reef communities, low temperature can also trigger bleaching with increased risk of mortality [17–19]. Climate-induced increases in SST are responsible for countless shifts in the structure and function of marine ecosystems [20] and has led to more frequent and severe mass coral bleaching events [21]. Tolerance to bleaching can be related to the thermal history of the environment [22] and habitat [23] but it is commonly attributed to the physiological thresholds and acclimation capacity of the coral host and their associated Symbiodiniaceae community [24].

The coral host and its symbionts may face physiological impairment and increasing risks of mortality during periods of sustained stress [25]. The molecular pathways involved in triggering, dealing with, and recovering from bleaching have been largely studied, however the responses are variable, and many questions remain [26]. Two important responses from the Symbiodiniaceae algae facing heat stress conditions are the increase in reactive oxygen species (ROS) production and changes in photosynthesis [27, 28]. In eukaryotic cells, ROS are produced by mitochondria, peroxisomes, and chloroplasts [29]. Despite being used as a signaling mechanism [30], increased ROS concentrations may cause oxidative damage to cellular components (e.g. nucleic acids, lipids, proteins and enzymes). Oxidative stress situation occurs when the production of ROS extrapolates the mitigating capacity of enzymatic (e.g., superoxide dismutase, ascorbate peroxidase and catalase) and non-enzymatic (e.g., ascorbate, glutathione, carotenoids, tocopherols) antioxidant compounds [31]. During stress, to relieve the reductive pressure on the chloroplast electron transport chain and balance the proportions of ATP and NADPH, electrons flow to oxygen instead of NADP+, and consequently the production of ROS increases [32, 33]. On the molecular level, ROS damages and hinders the repair of the D1 protein on photosystem II (PSII), damages the thylakoid membrane, and alters the activity of ribulose 1,5-bisphosphate carboxylase/oxygenase (Rubisco) that may lead to photosynthetic impairment and enzyme dysfunction [34, 35]. The suggestion that the susceptibility to thermal stress might be associated to the physiological response of the Symbiodiniaceae community associated with the coral resulted in many studies approaching the components of the symbiosis together and individually, using established cultures and freshly isolated Symbiodiniaceae cells. With few exceptions [36–38] studies report on the behavior of the microalgae populations, not considering the single cell response. Here we investigate the physiological response of three cultured Symbiodiniaceae strains facing a stepwise temperature increase. End points to measure the heat stress were cell size, chlorophyll fluorescence, photosynthetic performance, and single cell ROS production after 4 h and 72 h of exposure to the final testing temperature. We hypothesize an increased ROS production and impaired photosynthetic activity following the heat stress and that the level of response would differ among genetically different strains.

## Methods

### Symbiodiniaceae cultures

Three Symbiodiniaceae cultures previously isolated in 2012 from the coral *Mussismilia braziliensis* from the Abrolhos Reef Bank, the largest and most diverse reef system of the Southwestern Atlantic Ocean—Parcel dos Abrolhos, 16°40’S, 19°40’S; 039°10’W, 037°20’W, 2 to 6 m depth—were used in this study [39]. For this study no permits were required to access the field site because the cultures had been previously isolated and maintained as part of the microalgae culture collection hosted by our laboratory. These strains were originally published with the codes 103C3, 103C5 and 043D10 [39], and are herein coded CCMR0093, CCMR0095 and CCMR0100, respectively. The strains belong to the genera *Symbiodinium* (CCMR0095 and CCMR0100) and *Cladocopium* (CCMR0093), hereafter *S*. CCMR0095, *S*. CCMR0100, and *C*. CCMR0093. The cultures are maintained in the Culture Collection of Microalgae at the Federal University of Rio de Janeiro (CCMR) in f/2 medium [40] prepared with seawater from the Abrolhos Bank, salinity 35, pH 8.2, at 80 μmol photons m^-2^ s^-1^ (measured on the outer surface of the flasks) provided by cool light fluorescent tubes, and a 12:12h light:dark cycle. Cultures were maintained by successive transfers to fresh medium at approximately monthly intervals.

### Haplotype network analysis

ITS 2 sequences of the three Symbiodiniaceae strains used in this study (KJ189564, KJ189555, KJ189559) [39] were revisited to update and refine their taxonomic relationships using a haplotype network approach. These sequences were analyzed together with 52 ITS 2 sequences from Symbiodiniaceae from the South-Western Atlantic (36 sequences), the Caribbean (3 sequences), and the Pacific (7 sequences), plus 2 sequences from Panama and 1 from Bermuda (with no detailed geographic designation) [24, 41–46] and 3 sequences with no geographic designation deposited in GenBank (S1 Dataset). These sequences were organized in two sets, one for *Symbiodinium* (21 sequences) and one for *Cladocopium* (30 sequences), which were analyzed in parallel. A local BLASTn search was run comparing all sequences from each set against the SymPortal intra genomic ITS 2 variants database (www.symportal.org) [47] and their identity was recorded. In the case of sequences returning two or more hits with 100% identity, the one with the lowest E-value was selected for the downstream analysis (S1 Dataset). Only sequences with 100% identity were attributed a taxonomic ID relating them to a specific lineage (ITS 2-type) in SymPortal. Two sequences of *Cladocopium* (ITS 2-types C40c and C91h) were included in the dataset due to their high frequency among the best hits obtained from BLAST searches. The sequences were aligned using Clustal W and their size manually trimmed to the smallest one on both ends using Mega X [48]. The sequences were manually pairwise compared to identify indels and to assign them to specific haplotypes (i.e. consensus of 100% similar sequences). Indels were used as informative sites to build the network. Then, an estimate of genetic distances among the haplotypes was done using the Kimura 2-parameter substitution model [49] to check whether the haplotype designation was sound (i.e. 1 ≥ genetic distance > 0). Two haplotype networks (one for *Symbiodinium* and one for *Cladocopium*) were built using the Pegas package [50] in R v. 3.5.0 (R Core Team, 2018), with the TCS algorithm (parsimony network), considering each indels as informative sites as they bring relevant information in such a diverse DNA region as the ITS 2 [45]. A Templeton’s significance cutoff value [51] of 0.95 was used as a measure of significant linkage support. In the final network, groups of haplotypes were color-coded based on their closest BLASTn hits in the SymPortal database using Procreate illustration application (version 5.3.4).

### Experimental design

**Fig 1:**
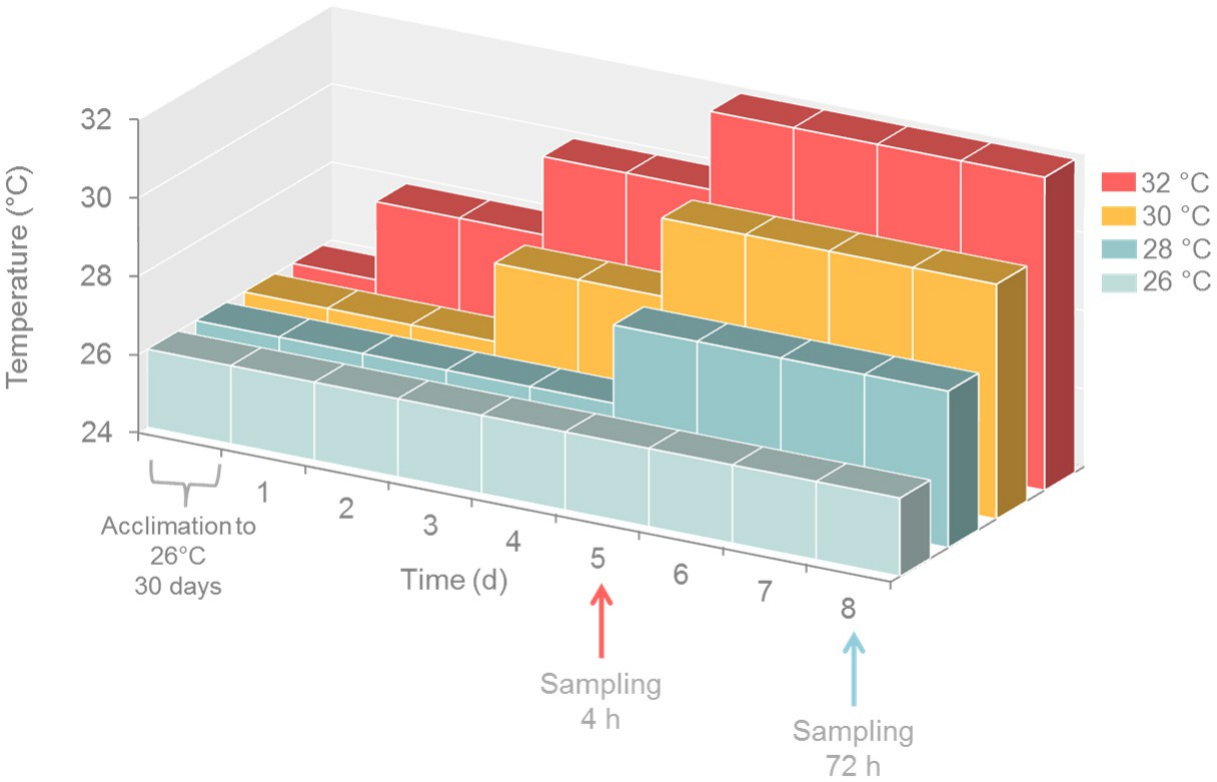
Changes in temperature over the course of the experiment exposing Symbiodiniaceae cultures to heat stress. Parent cultures were acclimated for 30 days at 26°C. At the start of the experiment parent cultures were aliquoted in 250 mL triplicate bottles for each temperature and were subjected to increases in steps of 2°C every second day. After 4 h and 72 h of exposure to the final temperature, the photosynthetic parameters were measured, and samples were taken for ROS production estimates and flow cytometric characterization (see S2 Fig).

The Symbiodiniaceae cultures were exposed to a stepwise increase in temperature within the range of 26 to 32°C (Fig 1). The low end of the range represents the modal historical temperature for the Abrolhos Bank area, whereas the high-end matches temperatures registered during the TGBE in the same area ([52] and S2 Fig) and an extreme temperature. The Photosynthetic Photon Flux Density (PPFD) was 80 μmol photons m^-2^ s^-1^ with a 12 h: 12 h light: dark period. This PPFD is within the range measured on roofs and walls of mushroom-like pinnacles characteristic of Abrolhos and near the bottom (S1 Fig). The Symbiodiniaceae physiological behavior was evaluated by flow cytometric-derived measurements of ROS production, morphological characterization, chlorophyll content, and maximum and effective quantum yields measured with Pulse Amplitude Modulated (PAM) fluorometry (diving-PAM, Walz, Effeldricht, Germany).

Parent cultures of the three Symbiodiniaceae strains were acclimated to 26°C for one month, while other culture conditions were kept as above. On the thirtieth day, the cell density of the cultures was adjusted to ca. 4.000 cells mL^-1^ by diluting them with fresh f/2 medium. The parent culture of each strain was aliquoted in 12 experimental units of 150 mL each in borosilicate 250 mL Erlenmeyer and kept at 26°C for 48h. Then, one set of triplicate vials of each strain was moved to an incubator at 28°C, whereas the others remained at 26°C. After another period of 48 h, another set of triplicate bottles was moved to 28°C, whereas the set that was at 28°C was moved to 30°C. This was repeated after 48 h, by moving another set of triplicate bottles from 26°C to 28°C, the one that was at 28°C was moved to 30°C, and the one at 30°C was moved to 32°C. One set of triplicate bottles (control) was kept at 26°C all the time. The physiological measurements were made in two moments: (i) 4 h and (ii) 72 h after the exposure of the cultures to their final experimental temperature (Fig 1 and S2 Fig).

### Reactive oxygen species measurements

Production of ROS was measured on a single-cell basis by means of the ROS-specific fluorescent probe 2’7’- dichlorodihydrofluorescein diacetate (H_2_DCFDA, Thermo Scientific D399) [53] coupled with detection by flow cytometry (BD Accuri C6). Upon cellular uptake, 2’7’- dichlorodihydrofluorescein (H_2_DCF) is formed from the cleavage of H_2_DCFDA, which is in turn oxidized to the highly fluorescent green compound dichlorofluorescein (DCF) in the presence of ROS (except by oxygen singlet). Aliquots of 15 mL were collected from each Symbiodiniaceae culture as explained above. These aliquots were subjected to one sonication step (10 s, 1 s pulses, 20% amplitude, Cole Parmer (CPX 130 sonicator, 3 mm probe) to loosen cell clamps. Sub-aliquots of 500 μL were transferred to 1.5 mL microtubes. Cells were analyzed in a Accuri C6 flow cytometer (Beckman Coulter, CA) equipped with a blue laser (488 nm, 20 mW) at a sample aspiration rate of 66 μL min^-1^ for 2 min. Green (530/30 nm band pass filter) and red (>670 nm long pass, 90% neutral density attenuation filter) fluorescence and forward-scattering light signals were collected. These measurements correspond to non-stained samples. Afterwards, 1 μL of a 10 mM solution of H_2_DCFDA in DMSO was added to each tube, followed by manual mixing and incubation at the experimental temperature and light regimes for 30 min. After the incubation, cells were again analyzed by flow cytometry. The green fluorescence signal of this second flow cytometric analysis was used as a measurement of relative ROS production.

### Other cytometric attributes

Cytometric data collected from non-stained Symbiodiniaceae cells were used to characterize cell attributes of the three strains. The population of Symbiodiniaceae cells was discriminated in a forward-scattered light vs. red fluorescence using the software supplied by the manufacturer of the flow cytometer (Accuri C6 software). Forward scattering (FSC-H) and red fluorescence (FL3-H) pulse height were used as proxies for relative cell size and chlorophyll content, respectively. Data was exported and handled in MSExcel.

### Quantum yield of PSII

The photosynthetic efficiency of Symbiodiniaceae cultures was measured in a pulse amplitude modulated fluorometer (diving-PAM, Walz, Effeldricht, Germany), equipped with a blue LED (470nm) [54]. The experimental units were homogenized manually, and an aliquot of 10 mL was sampled from each flask and placed into a scintillation vial. The fiber optics and the scintillation vials were placed in a metal clip support to maintain a fixed distance between them. The fiber optics was externally placed under the bottom of the vial for measurements. The effective quantum yield of the photochemical conversion [F_PSII_ = (F’_m_- F)/F’_m_] was measured immediately after samples were taken from each experimental unit 4 h and at 72 h after exposures to the higher temperature in each treatment (S2 Fig). Light-adapted fluorescence yield (F) corresponds to fluorescence of the cultures at regular growth light condition provided by cool light fluorescent tubes (80 μmol photons m^-2^ s^-1^), and maximum light-adapted fluorescence yield (F’_m_) was estimated after an 800 ms light saturating pulse of 6000 μmol photons m^-2^ s^-1^. Thereafter, samples were placed in the dark for 20 min to determine the maximum quantum yield of the PSII (F_v_/F_m_). The minimal fluorescence level (F_0_) was detected under the modulated measuring light of the PAM (a week pulsed light, <1 μmol photons m^-2^ s^-1^), whereas the maximum fluorescence level (F_m_) was obtained with a saturating pulse of light (800 ms pulse of 6,000 μmol photons m^-2^ s^-1^). Variable fluorescence (F_v_) was calculated from (F_m_−F_0_) and the maximum quantum efficiency of PSII photochemistry was obtained from the ratio F_v_/F_m_. The non-photochemical coefficient (NPQ) was calculated from (F_m_-F’_m_)/F_m_, where F_m_ corresponds to the maximum fluorescence obtained from dark-adapted samples, and F’_m_ from light adapted samples. The diving-PAM settings were measuring light (ML) = 4, saturating pulse intensity (ST) = 8, saturating width (SW) = 0.8s, gain (G) = 2, and damping (D) = 1.

### Statistical analysis

The response of the Symbiodiniaceae populations to thermal stress was assessed independently for each isolate. Linear mixed-effect models were used to evaluate the effects of temperature and recovery time on cytometric (FSCH, FL1-H, FL3-H) and photosynthetic (F_v_/F_m_, Y(II), NPQ) response attributes [55]. Cytometric data was evaluated on a log10 basis. Temperature was treated as a continuous variable to assess population tendencies with increasing temperatures. Exposure time was treated as a categorical variable and a random term was used to account for repeated measures at the same experimental units i.e. flasks (on 4 h and 72 h). Shapiro-Wilks tests were used to evaluate normality of the residuals, and Levene’s tests for homoscedasticity over each explanatory variable [56]. When necessary, either generalized linear mixed models (with Gaussian error distribution and logarithmic link function) or data transformation (power functions) were used to ensure the adequacy of the modeling strategy [57] (see S4 Dataset).

Moreover, pairwise comparisons were used to look for differences in specific levels of either exposure temperature or exposure time. Differences between exposure times in each exposure temperature were checked by paired t-tests. Complementary, assuming 26°C as a reference temperature, t-tests were used to check for statistically different exposure temperatures in each exposure time. Independent comparisons were made for temperatures 4 h and 72 h after temperature increase. For both explanatory variables, results were adjusted for multiple comparisons with a 10% false-discovery rate [58]. All statistical analysis was done on R, version 4.1.2 [59].

## Results

### Haplotype networks

**Fig 2:**
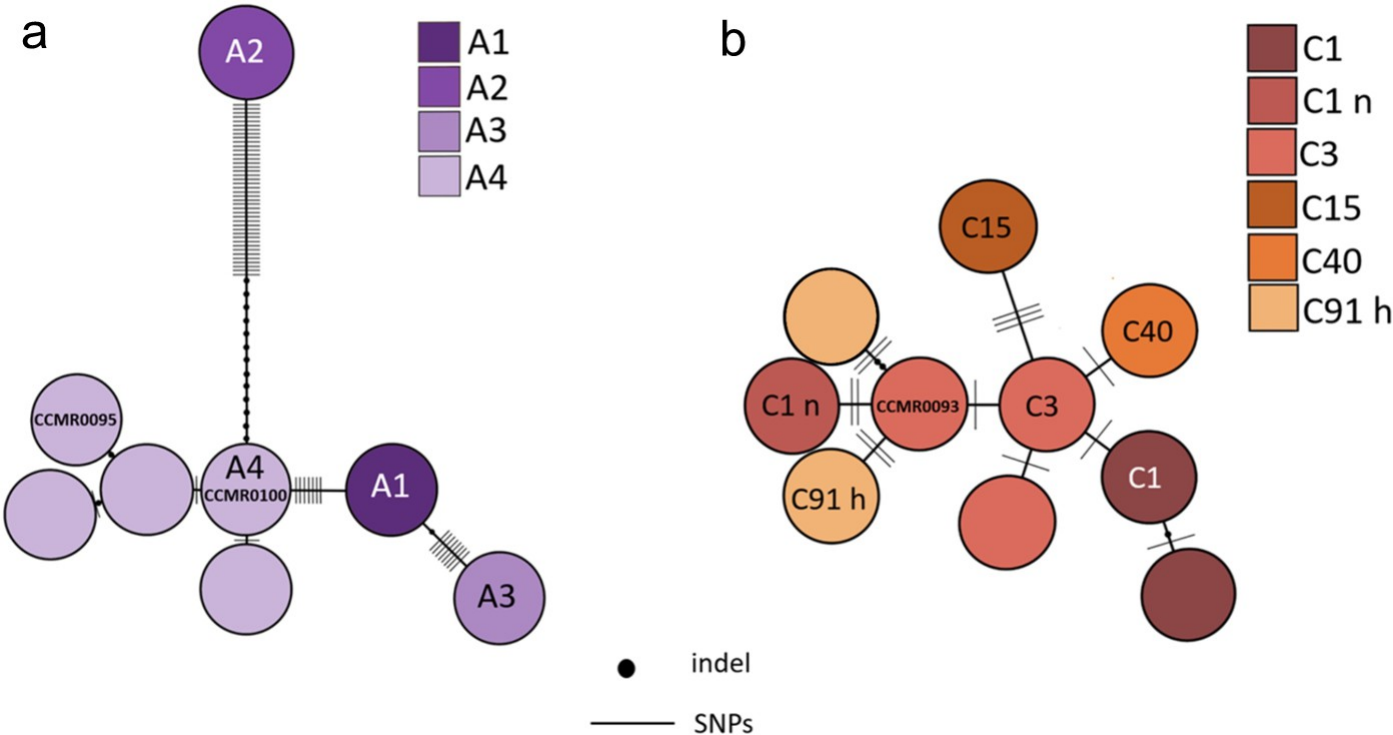
Minimum haplotype network for the Symbiodiniaceae ITS 2 unique sequences. Hatch marks on the lines represent a single nucleotide polymorphism (SNPs) and black dots represent indel sites. Haplotypes that did not match any previously defined ITS 2 types are color labeled according to the best hit identity obtained using Blastn. (a) *Symbiodinium* (b) *Cladocopium*.

A total of 8 and 10 haplotypes were recovered from the original sequence data set for *Symbiodinium* and *Cladocopium*, respectively (S1 Dataset). Networks were built on 213 and 217bp long sequence alignments for the *Symbiodinium* and *Cladocopium* datasets, respectively. Among the 8 haplotypes of *Symbiodinium*, four showed 100% identity to the ITS 2-types A1, A2, A3 and A4 (Fig 2a). The other four haplotypes had 1 to 4 mutational steps among them and formed a cluster with 99.4% to 99.6% identity with the ITS 2-type A4. Overall, the eight *Symbiodinium* ITS 2 types had between 7 and 55 mutational steps among them. The strain *S*. CCMR0100 was identified as ITS 2 type A4 with 100% identity, whereas the strain *S*. CCMR0095 resulted as a derivative haplotype from A4 by two mutational steps (1 indel and 1 SNP). Conversely, the *Cladocopium* network showed less clear clustering (Fig 2b). The whole network (10 haplotypes) displayed from 1 to 4 mutational steps. Six haplotypes of this network had 100% sequence identity with ITS 2 types C1, C1n, C3, C15, C40, and C91h. The other four haplotypes were closely related to C1 and C3, albeit with more than 99.8% identity. The strain *C*. CCMR0093 resulted in 99.8% identity with the ITS 2 type C3, with two mutational steps (SNPs). All posterior probabilities of both networks were >0.95.

### ROS production

**Fig 3:**
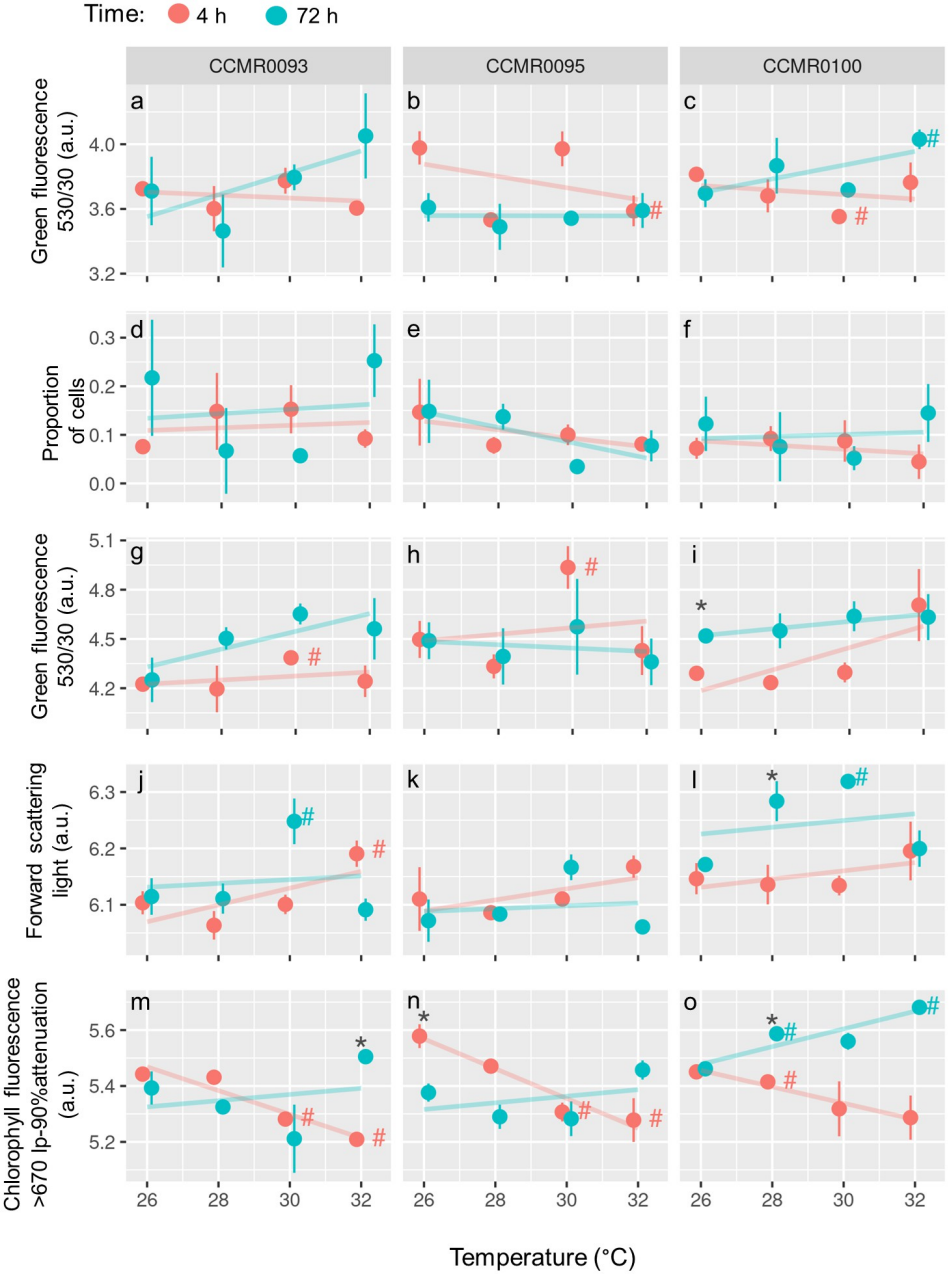
Responses of three Symbiodiniaceae cultures (*Cladocopium* sp. CCMR 0093; *Symbiodinium* sp. CCMR0095; *Symbiodinium* sp. CCMR0100) to thermal stress. Each column of graphs represents one Symbiodiniaceae strain. Rows represent each variable measured using flow cytometry (a to c): average green fluorescence of the whole symbiont cell population stained with DCF; (d to f): proportion of ROS-positive cells i.e. cells above the green fluorescence threshold for DCF (see S2 Fig); (g to i): average green fluorescence of the ROS-positive cell population stained with DCF; (j to l): relative cell size expressed as average forward scattering light (FSC) signal of the whole non-stained symbiont cell population; (m to o): average chlorophyll fluorescence of the whole non-stained symbiont cell population. (#): Significant difference compared to the control group at 26°C (t-test; adjusted.p<0.1); (*): Significant difference compared to the same temperature and culture at 4 h and 72 h (t-test; adjusted.p<0.1). a.u.: arbitrary units.

Average ROS production considering the whole cell population showed no clear trend among temperatures and times of exposure. A significant, positive effect of exposure time was observed for strain *S*. CCMR0100 (F = 5.9; p = 0.0340), indicating higher levels of ROS production at 72 h after the temperature increase (Fig 3a–3c, S4 Dataset). For isolate *S*. CCMR0095, the opposite was observed with a significant negative effect of exposure time on green fluorescence (F = 11.2; p = 0.0070) (S4 Dataset). Significant differences between individual high temperature treatments and modal Abrolhos temperature (26°C) control were found at 30 and 32°C for strain *S*. CCMR0100 and 32°C for strain *S*. CCMR0095 (Fig 3a–3c).

The cytometric single-cell analysis of H_2_DCFDA-stained Symbiodiniaceae cells showed sub-populations with distinct levels of ROS production (S3 Fig), one low and the other one high in green fluorescence. On average, between 5.2 and 25.3% of cells stained with H_2_DCFDA had their green fluorescence increased above the threshold (high ROS production, ROSh) (Fig 3d–3f). For the three Symbiodiniaceae strains studied, neither temperature nor exposure time had a significant effect on the percentage of ROSh subpopulation (S4 Dataset). The average ROS production of the ROSh population (Fig 3g–3i) was on average six times higher than the average for the whole population. Temperature and exposure time had positive significant effects on ROS production in the ROSh population for isolates *C*. CCMR0093 (F = 6.2;p = 0.003 and F = 27.1; p = 0.000, respectively) and *S*. CCMR0100 (F = 11.3; p = 0.007 and F = 21.7; p = 0.001, respectively), indicating a tendency of higher ROS production on higher temperatures and 72 h after the temperature increase (S4 Dataset). Pairwise comparisons showed significant increase in ROS production compared to the 26°C reference temperature for strains *C*. CCMR0093 (30°C at 4 h) and *S*. CCMR0095 (30°C at 4 h).

### Relative cell size and chlorophyll fluorescence

For the Symbiodiniaceae strain *S*. CCMR0100, exposure time was a significant factor (F = 17.0; p = 0.002) influencing relative cell size (measured as forward scattering light), indicating larger cell sizes 72 h after the temperature increase. The linear mixed-effect models result indicated no significant effect of temperature or exposure time for strains *C*. CCMR0093 and *S*. CCMR0095. On the other hand, pairwise comparisons revealed a significant increase in average cell size for strains *C*. CCMR0093 subjected for 4 h to 32°C (22% higher FSC-H) and 72 h to 30°C (36% higher FSC-H), and *S*. CCMR0100 subjected for 72 h at 30°C (40% higher FSC) compared to the respective controls subjected to 26°C (Fig 3j–3l). Exposure time had a significant positive effect on the relative chlorophyll cellular content in Symbiodiniaceae *S*. CCMR0100 (F = 32.3; p = 0.000), whereas temperature did not present a clear tendency (Fig 3m–3o; S4 Dataset). For the Symbiodiniaceae strains *C*. CCMR0093 and *S*. CCMR0095, we did not observe a significant effect of temperature and exposure time on the cellular chlorophyll content (S4 Dataset) despite a significant decrease in relation to 26°C observed in pairwise comparisons in 30°C and 32°C treatments at 4 h exposure.

### Photochemical performance

**Fig 4:**
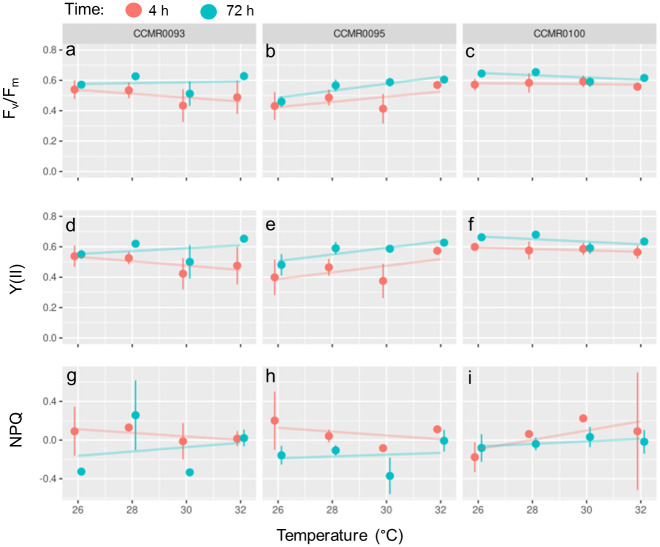
Photochemical performance obtained from PAM fluorometer analysis. Strains are represented in columns, and variables as rows. a to c: maximum quantum yield measurements; d to f: Effective quantum yields; and g to i: NPQ. The colors indicate the moment of sampling (4 h, red; 72 h, blue).

The lowest value of F_v_/F_m_ was 0.356, observed for the strain *C*. CCMR0093 after being exposed to 30°C for 4 h, whereas the highest value (0.672) was observed in the control temperature (26°C) of the strain *S*. CCMR0100 after 72 h. Y(II) values ranged from 0.274 for *S*. CCMR0095 exposed for 4 h to 30°C, to 0.688 for *S*. CCMR0100 after 72 h of exposure to 28°C. Our results indicate a significant effect of exposure time on F_v_/F_m_ for the three Symbiodiniaceae isolates (S4 Dataset). The same pattern was observed for Y(II), in which yields were consistently higher at 72 h than at 4 h after temperature increase (Fig 4, S3 Dataset). For the three Symbiodiniaceae isolates, the effect of exposure time was more pronounced than temperature, and only for isolate *S*. CCMR0095, exposure temperature had a significant effect on F_v_/F_m_ and Y(II) (S4 Dataset). Overall, observed NPQ values were low, with averages oscillating around zero. Linear mixed model result indicated a negative, albeit small, effect of time on NPQ for strains *C*. CCMR0093 (F = 5.1; p = 0.044) and *S*. CCMR0095 (F = 18.1; p = 0.001) with no detectable pairwise differences between treatments across temperature or in relation to the reference temperature of 26°C.

## Discussion

We herein subjected cultures of three Symbiodiniaceae strains isolated from the Abrolhos reefs to a stepwise increase in temperature within the limits of their natural environment and observed an overall thermotolerance, which may have implications to Abrolhos coral’s resilience. Monoculture and freshly isolated populations of Symbiodiniaceae cells have been used to build a comprehensive picture of the temperature effects on physiological thresholds of the symbiont, given the reported differences in susceptibility within the group [3, 53]. Despite limitations in extrapolating results from experiments with *ex hospite* Symbiodiniaceae to the natural habitat [60, 61], such approaches make up an important part of the strategy to understand key mechanisms related to the microalgae diversity and responses to environmental stressors [62–64]. This is especially true due to the possibility of studying and comparing different genotypes. *In situ* observation of *in hospite* symbionts or handling of freshly isolated natural populations of symbionts in experimental setups may avoid unwanted loss or changes in certain traits but, on the other hand, reflect only average responses of mixed communities of Symbiodiniaceae [65].

The haplotype network based on the SymPortal database confirmed the identity of the *S*. CCMR0100 as type A4 [39], whereas strains *S*. CCMR0095 and *C*. CCMR0093 were identified as derivative sequences from types A4 and C3 by two and one mutational step, respectively. Such differences should be considered when comparing metabolic and physiological traits among the strains. *C*. CCMR0093 and *S*. CCMR0095 haplotype sequences showed to be unique in the database (SymPortal) which may indicate they belong to hitherto undescribed species of Symbiodiniaceae. The genus *Cladocopium* (former clade C) is the most diverse and widely distributed depth, latitude and hostwise, whereas *Symbiodinium* (former clade A) is commonly associated with shallow water environments [66]. The different response to thermal stress observed in this study between *Symbiodinium* strains (*S*. CCMR0100 vs. *S*. CCMR0095) is in line with a large range of variation on thermal tolerance within this genus [67].

The increase in temperature damages the photosynthetic apparatus [8, 68, 69] that can result in photoinhibition and increased production of ROS which may lead to the cell apoptosis and disruption of the symbiosis [70]. The stepwise temperature increase of our experimental design may have rendered the cells some resistance to the stress due to a period of acclimation. Normally, laboratory setups subject Symbiodiniaceae cells to instant transfers to higher temperatures than controls and with a much steeper ramp [71–74] compared to our approach. In our results, the average ROS production on a population basis was not strongly influenced by high temperatures as no overall effect was observed. The only significant increase in relation to control was observed for strain S. CCMR0100 at 32°C where ROS production doubled. This 2-fold increase is of the same order of magnitude observed for the highest ROS production following thermal stresses from 26°C to 30–35°C in Symbiodiniaceae reported elsewhere [75]. Such low sensitivity to temperature increase in our Symbiodiniaceae cultures may be related to the relatively low level of light intensity (80 μmol photons m^-2^ s^-1^) used in the experiments [11]. This light intensity is typical of large areas of the Abrolhos reefs near the bottom and under the top of the mushroom-shaped pinnacles characteristic of this turbid reef system [76] (S1 Fig). As mentioned above, the rate of temperature increase may also influence the response. For example, an increase from 27 to 32°C in a 4 h period elicited a ca. 10-fold increase in the average cellular ROS production of Symbiodiniaceae population harvested from a coral [73]. Comparatively, the same temperature change in our experiments, mimicking real conditions, was achieved in ca. 6 d.

Microalgal cultures and natural populations present heterogeneity among individual cells regarding morphology, metabolism and cellular contents e.g. pigments and nucleic acids [77]. Most studies on cultured Symbiodiniaceae are on the population level, whereas single cell-based approaches, as we used here, are less frequent [36–38]. Flow cytometric methods have the advantage of allowing high throughput measurements in many live cells [78]. One interesting result in our experiments was the systematic intra-population variability in ROS production in the Symbiodiniaceae cultures, with a bimodal distribution for the variable green fluorescence in the H_2_DCFDA stained cells (S3 Fig). Assuming that natural populations of *in hospite* Symbiodiniaceae also present this intra-population variation in ROS production, the ability to discriminate the status of single cells opens up the possibilities to explore the physiological status of this alga in populations of freshly isolated cells. One reason for this intrapopulation variation is the fact that cells do not divide in an absolute synchronous way, which result in cells of different ages (phase of the cell cycle) [77]. Also, the intrapopulational response may be influenced by differences in the abundance and composition of the bacteria community associated to the phycosphere among cells [79, 80]. Symbiodiniaceae cultures commonly have co-isolated bacteria that enhance population growth rates, and cellular health and viability, and it has been also demonstrated that specific bacteria taxa (e.g. *Muricauda* sp.) are able to mitigate the effect of oxidative stress in these dinoflagellates by producing antioxidant carotenoids [80–82]. It is reasonable to assume that this process influenced our cultures as they are not axenic, as aren’t the majority of Symbiodiniaceae cultures reported elsewhere [74].

The increase of cell size observed for the strains S. CCMR0100 exposed to high temperature may be related to a high demand of energy for acclimation which would lead to the arrest of other cellular processes. Heat stress damages DNA and leads to cell cycle arrest in eukaryotic cells at pathway checkpoints that control the timing of cell cycle progression and detect such damages, resulting in prevention of replication of damaged DNA templates which leads to the accumulation of abnormally large cells in the G1 phase [77, 83].

Heat stress may result in contrasting behaviors on chlorophyll levels in Symbiodiniaceae [15, 84]. The consistent decreasing tendency in chlorophyll fluorescence after 4 h of exposure for all strains tested here could be related to the degradation of such molecules or changes related to a photoacclimation response, indicative of mild stress to the photosynthetic apparatus. Strychar & Sammarco [85] showed the same pattern for Symbiodiniaceae photopigments associated with three different coral species through a range of temperature (28°C—34°C) similar to the one we subjected our strains, but after 48 h of exposure. The return of chlorophyll fluorescence to initial levels after ca. 2,8 d (the time lapse between 4 h and 72 h) indicates that mechanisms of photoacclimation were activated. This plasticity of the Symbiodiniaceae photosystem facing heat stress may explain the wide optimum range of photosynthesis between 26°C and 32°C for these symbionts [86, 87]. An interesting approach would be the analyses of the carotenoids pigments, especially the ones that compose the xanthophyll cycle, as these molecules are the ones that dissipate the most energy for their proximity to the light harvesting complex of PSII (LHCII) [88].

In terms of photosynthetic efficiency, none of the strains tested had a negative effect on F_v_/F_m_ neither (YII). Indeed, in one strain (*S*. CCMR0095) a positive effect on these photosynthetic parameters was observed both for an increase in temperature and time of exposure. Symbiodiniaceae cells or corals subjected to a combination of high temperature and high PPFD (e.g. 600–1500 μmol photons m^-2^ s^-1^) normally show a reduction in photosynthetic efficiency [33, 71]. The light condition, temperature ramp and duration of the heat stress that we used do not seem to have elicited loss of photosynthetic efficiency nor the activation of non-photochemical mechanisms in the cells. This fact highlights a generalized thermotolerance as well as some physiological difference between closely related *Symbiodinium* sp. (ITS 2 type A4). Nevertheless, given that the ITS 2 marker may overestimate Symbiodiniaceae diversity [65] taxonomic refinements and more physiological studies of Symbiodiniaceae strains from Abrolhos bank corals will greatly help our understanding of their function in this system. Also, as *in hospite* symbiont cells may differ morphologically and physiologically from cultured strains [61] measuring such photosynthetic parameters in freshly isolated symbionts are relevant to complete the picture.

## Conclusion

Our results indicate that the three Symbiodiniaceae cultures isolated from Abrolhos corals were not negatively impacted by the stepwise increase in temperature within a range observed in the reefs. The recovery on chlorophyll cellular content and quantum yields following exposure to high temperatures, without an exacerbated increase in ROS production across the experiments, suggests a tolerance of the cultures to the new temperature condition. Despite such overall similarities, intra-specific variability could be detected, likely reflecting the genetic differences among the strains. Furthermore, seldom-explored intra population variability was observed for all strains regarding ROS production, which highlights the importance of single-cell measurements in cultures and in freshly isolated cells for a more realistic picture at population level.

## Supporting information

S1 Appendix Description of how water and irradiance measurements were obtained at the field site. (DOCX)

S1 Fig Temperature and light measurements. One year temperature measurements (°C) obtained in Pedra de Leste site using an underwater monitoring sensor, at the Abrolhos Reef Bank (a). Column graphs presents the bimodal temperatures for this site during the specified period (b). Light measurements (μmol photons m^-2^ s^-1^) in different niches of the mushroom-shaped pinnacles (c).
(JPG)

S2 Fig Experiment timeline. Experimental design timeline Parent cultures were acclimated for 30 days at 26°C. At the start of the experiment an aliquote from the parent cultures was inoculated in fresh media to obtain the expected inicial cellular concentration, through dilution. From the new homogenate, parent cultures were aliquoted in triplicate bottles for each temperature and place at 26°C incubator. After 48h the first step increasing the temperature began. Following the increases in steps of 2°C every 48h, until they reached the final temperature of exposure. At 4 h and 72 h photosynthetic parameters were measured, and samples were taken for flow cytometric analusis and ROS production estimates.
(JPG)

S3 Fig Unstained and stained cells cytograms. Cytograms of forward-scatter (FSC-H) vs. green fluorescence (530/30 nm) showing populations of *Cladocopium* sp. CCMR0093 cells in culture after 4 h of exposure to 28°C. **(a)** Live symbiont cells in cultures with no addition of ROS-reactive fluorochrome H_2_DCFDA and **(b)** with 10 μM of the fluorochrome. The horizontal red line represents the green fluorescence threshold for non-stained cells and is set to the same level in both cytograms for comparison. In this example, a background of 2.6% of cells were observed above threshold in the non-stained culture whereas 23.9% of the cells displayed fluorescence above threshold in the H_2_DCFDA treated culture. Forward scatter and fluorescence values are in log scales. a.u.: arbitrary units.
(JPG)

S1 Dataset Initial Symbiodiniaceae ITS sequences used for the alignment file to be identified as unique haplotypes. Blast results of unique haplotypes of Symbiodinium and Cladocopium used to build the haplotype networks and their sequence information.
(XLSX)

S2 Dataset Raw date obtained from PAM fluorometer and flow cytometric analysis. (XLSX)

S3 Dataset Maximum and minimum values of F_v_/F_m_ and Y(II) obtained during the experiment. (XLSX)

S4 Dataset Statistical analysis results. (XLSX)

## Abbreviations

CCMR: Culture Collection of Microorganisms
ETR: Electron transport rate
FCM: Flow cytometry methods
PPFD: Photosynthetic photon flux density
PSI: Photosystem I
PSII: Photosystem II
ROS: Reactive oxygen species
SST: Sea surface temperature
TGBE: Third global bleaching event
UFRJ: Federal University of Rio de Janeiro
