# Supplementary material for: Physiological response of Symbiodiniaceae to thermal stress: Reactive oxygen species, photosynthesis, and relative cell size
Source: PLoS One. 2023 Aug 3;18(8):e0284717. doi: 10.1371/journal.pone.0284717 (PMC10399794; doi:10.1371/journal.pone.0284717)
Supplement: S1 Appendix — (DOCX) [file pone.0284717.s001.docx]

**S1. Text. Description of temperature and irradiance measurements in the field.** Water temperature was recorded continuously from January 2016 to March 2017 with HOBO sensors (HOBO Pendant® measurements.  Temperature/Light 8K Data Logger) installed in Pedra de Leste. Light irradiance measurements using the underwater Li-Cor (LI-COR Biosciences LI-1000 Datalogger) were taken by scuba diving with the sensor on the surface and on five sites (top, edge, roof, wall and bottom) in one mushroom pinnacle at Parcel dos Abrolhos, to map the light conditions.
