# Supplementary figures and images for: Physiological response of Symbiodiniaceae to thermal stress: Reactive oxygen species, photosynthesis, and relative cell size
Source: PLoS One. 2023 Aug 3;18(8):e0284717. doi: 10.1371/journal.pone.0284717 (PMC10399794; doi:10.1371/journal.pone.0284717)

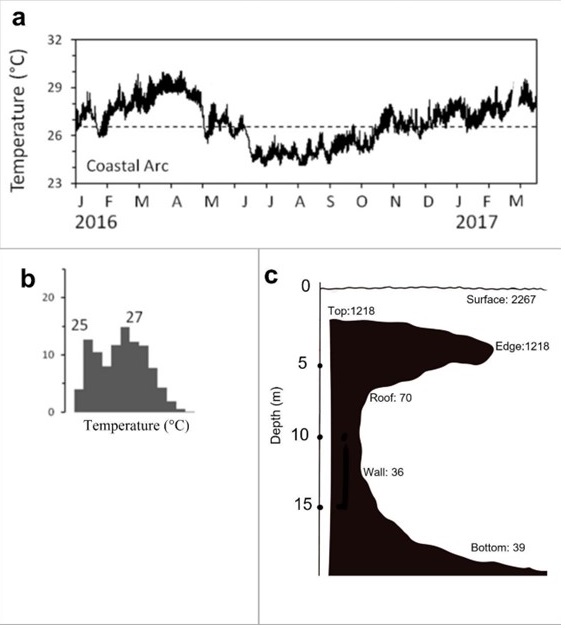

Supplement: S1 Fig — One year temperature measurements (°C) obtained in Pedra de Leste site using an underwater monitoring sensor, at the Abrolhos Reef Bank (a). Column graphs presents the bimodal temperatures for this site during the specified period (b). Light measurements (μmol photons m-2 s-1) in different niches of the mushroom-shaped pinnacles (c). (JPG) [file pone.0284717.s002.jpg]

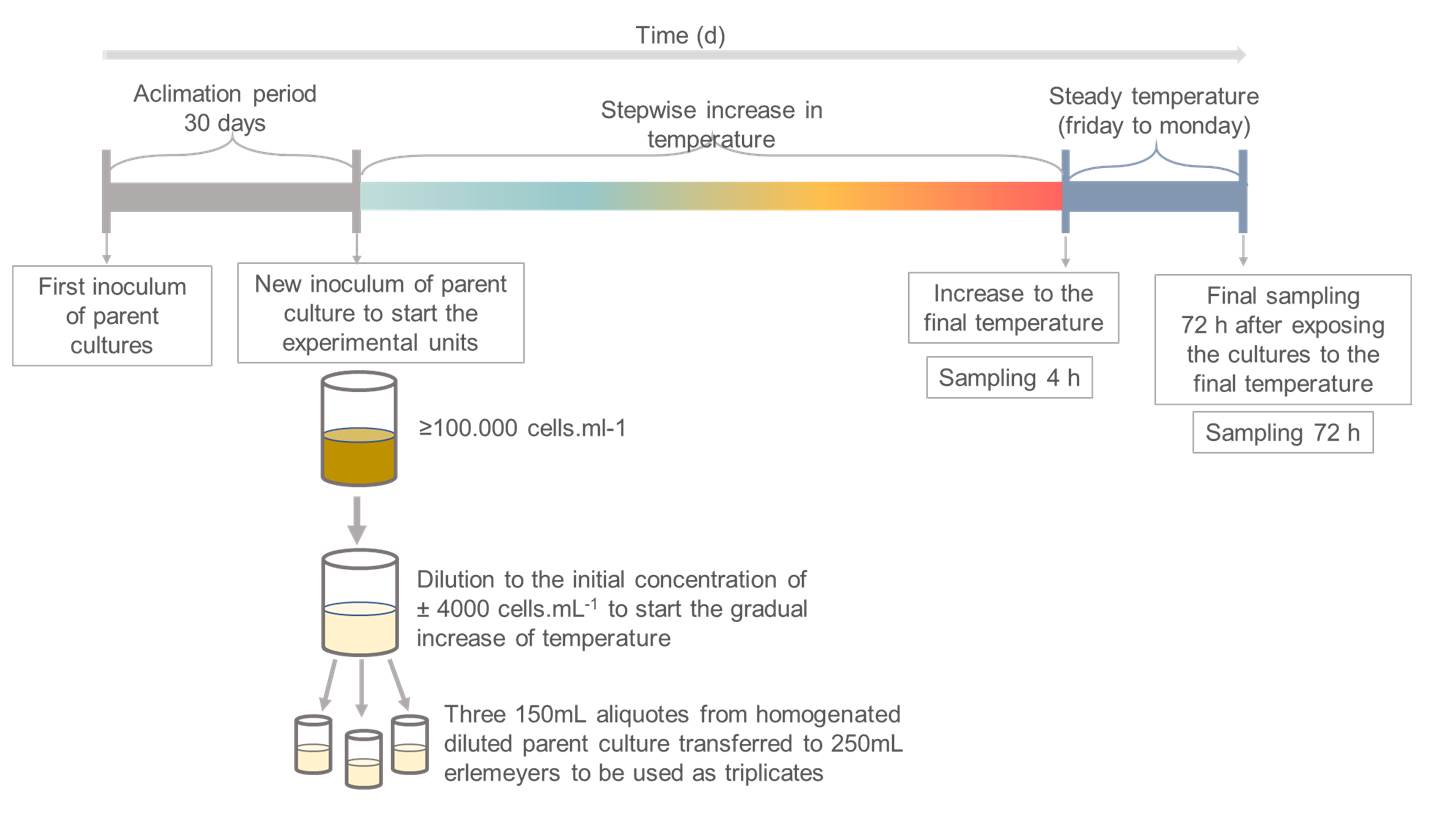

Supplement: S2 Fig — Experimental design timeline Parent cultures were acclimated for 30 days at 26°C. At the start of the experiment an aliquote from the parent cultures was inoculated in fresh media to obtain the expected inicial cellular concentration, through dilution. From the new homogenate, parent cultures were aliquoted in triplicate bottles for each temperature and place at 26°C incubator. After 48h the first step increasing the temperature began. Following the increases in steps of 2°C every 48h, until they reached the final temperature of exposure. At 4 h and 72 h photosynthetic parameters were measured, and samples were taken for flow cytometric analusis and ROS production estimates. (JPG) [file pone.0284717.s003.jpg]

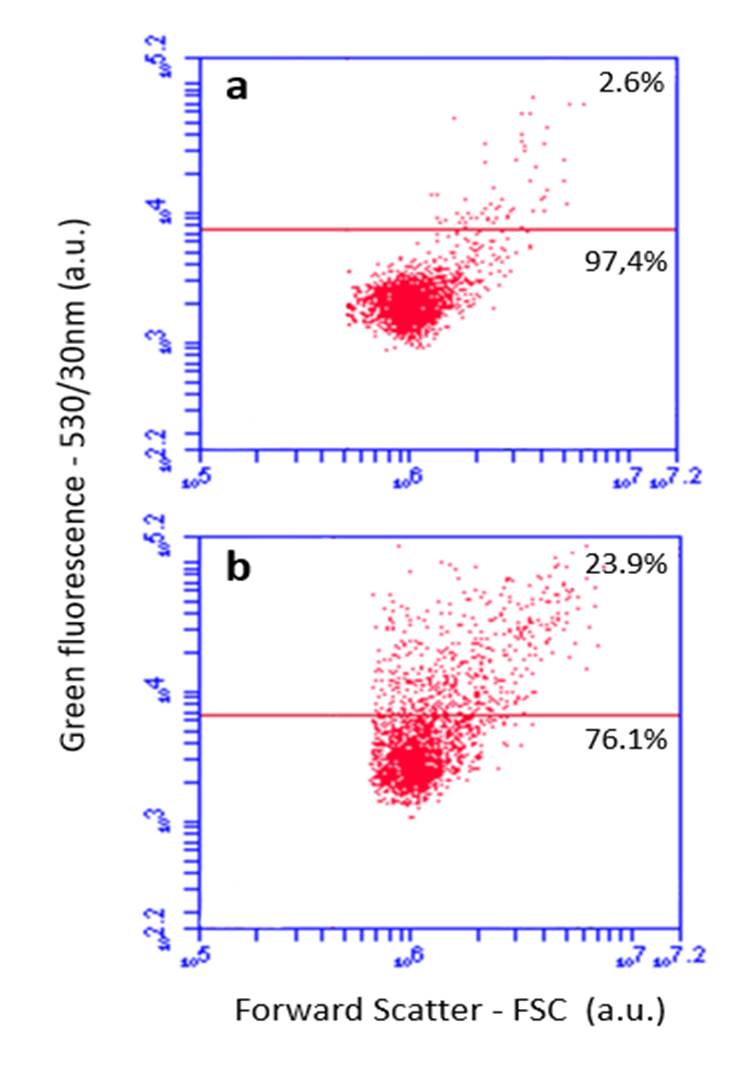

Supplement: S3 Fig — Cytograms of forward-scatter (FSC-H) vs. green fluorescence (530/30 nm) showing populations of Cladocopium sp. CCMR0093 cells in culture after 4 h of exposure to 28°C. (a) Live symbiont cells in cultures with no addition of ROS-reactive fluorochrome H2DCFDA and (b) with 10 μM of the fluorochrome. The horizontal red line represents the green fluorescence threshold for non-stained cells and is set to the same level in both cytograms for comparison. In this example, a background of 2.6% of cells were observed above threshold in the non-stained culture whereas 23.9% of the cells displayed fluorescence above threshold in the H2DCFDA treated culture. Forward scatter and fluorescence values are in log scales. a.u.: arbitrary units. (JPG) [file pone.0284717.s004.jpg]
